# Silent Circulation of Rift Valley Fever in Humans, Botswana, 2013–2014

**DOI:** 10.3201/eid2610.191837

**Published:** 2020-10

**Authors:** Claire E. Sanderson, Ferran Jori, Naazneen Moolla, Janusz T. Paweska, Nesredin Oumer, Kathleen A. Alexander

**Affiliations:** Virginia Tech, Blacksburg, Virginia, USA, and Chobe Research Institute, Center for African Resources:; Communities, Animals, and Land Use, Kasane, Botswana (C.E. Sanderson, K.A. Alexander);; Animals, Health, Territories, Risks, Ecosystems Unit, Université de Montpellier, Montpellier, France, and Botswana College of Agriculture, Gaborone, Botswana (F. Jori);; National Institute of Communicable Diseases, Sandringham-Johannesburg, South Africa (N. Moolla, J.T. Paweska);; University of the Witswatersrand, Johannesburg (J.T. Paweska);; Botswana Ministry of Health, Gaborone (N. Oumer).

**Keywords:** Aedes mosquitos, arboviruses, Botswana, Culex mosquitos, infectious disease transmission, livestock, One Health, pregnancy outcomes, public health, Rift Valley fever, viruses

## Abstract

We evaluated the prevalence of Rift Valley fever virus IgG and IgM in human serum samples (n = 1,276) collected in 2013–2014 in northern Botswana. Our findings provide evidence of active circulation of this virus in humans in the absence of clinical disease in this region.

The World Health Organization considers Rift Valley fever (RVF) a priority disease because of its substantial public health impact and the lack of available interventions to prevent and halt epidemics (*1*). RVF virus (RVFV) is primarily transmitted to animals through infected *Aedes* and *Culex* mosquitoes, while human transmission has been attributed to direct contact with the blood and tissues of RVFV-infected livestock. RVF outbreaks have been challenging to forecast because quiescent interepidemic or interepizootic years are irregularly interspersed with epizootic years. Both RVFV-infected mosquitoes and terrestrial mammals are postulated to play a role in virus maintenance during interepizootic years (*2*). 

In Botswana, RVFV exposure and infection dynamics are incompletely understood. Despite numerous large-scale RVF outbreaks across southern Africa being reported to the World Animal Health Information Database (https://www.oie.int/wahis_2/public/wahid.php/Wahidhome/Home), no outbreaks in people have been detected in Botswana, even though previous surveys have found serologic evidence of virus exposure in humans (1959, 1984–1986), African buffalo (*Syncerus caffer*), and domestic cattle (*3**–**5*). Thus far, according to the World Animal Health Information Database, RFV disease outbreaks in Botswana have only been reported in livestock (n = 4 outbreaks). It is presently unclear why low-level virus circulation has not been associated with detectable outbreaks in humans or how the virus is maintained during interepizootic years. Here, we evaluate archived human serum samples for evidence of RVFV-specific IgG and IgM and discuss the implications for public health in this region. 

## The Study 

We determined the historical occurrence of suspected and documented cases of RVF in the human population in Botswana by evaluating inpatient records from Kasane Primary Hospital (Chobe District, 1962–2019) and nationwide monthly outpatient data from all 17 districts (1985–2019). Human serum samples (n = 1,276; mean age 32 [SD +12], range 1–91; 2013–2014) were collected from government health facilities within the Chobe District and screened using a recombinant nucleocapsid IgG indirect ELISA (*6*), with positive samples confirmed by inhibition ELISA (*7*). We screened IgG-positive samples for IgM using IgM-capture ELISA (*8*). This research was conducted with permission from the Botswana Ministry of Health and the Virginia Tech Institutional Review Board (Permit #11–573). Figure was created in the open source statistical program R version 3.6.1 (https://www.r-project.org) using *ggplot2* (https://ggplot2.tidyverse.org). 

**Figure:**
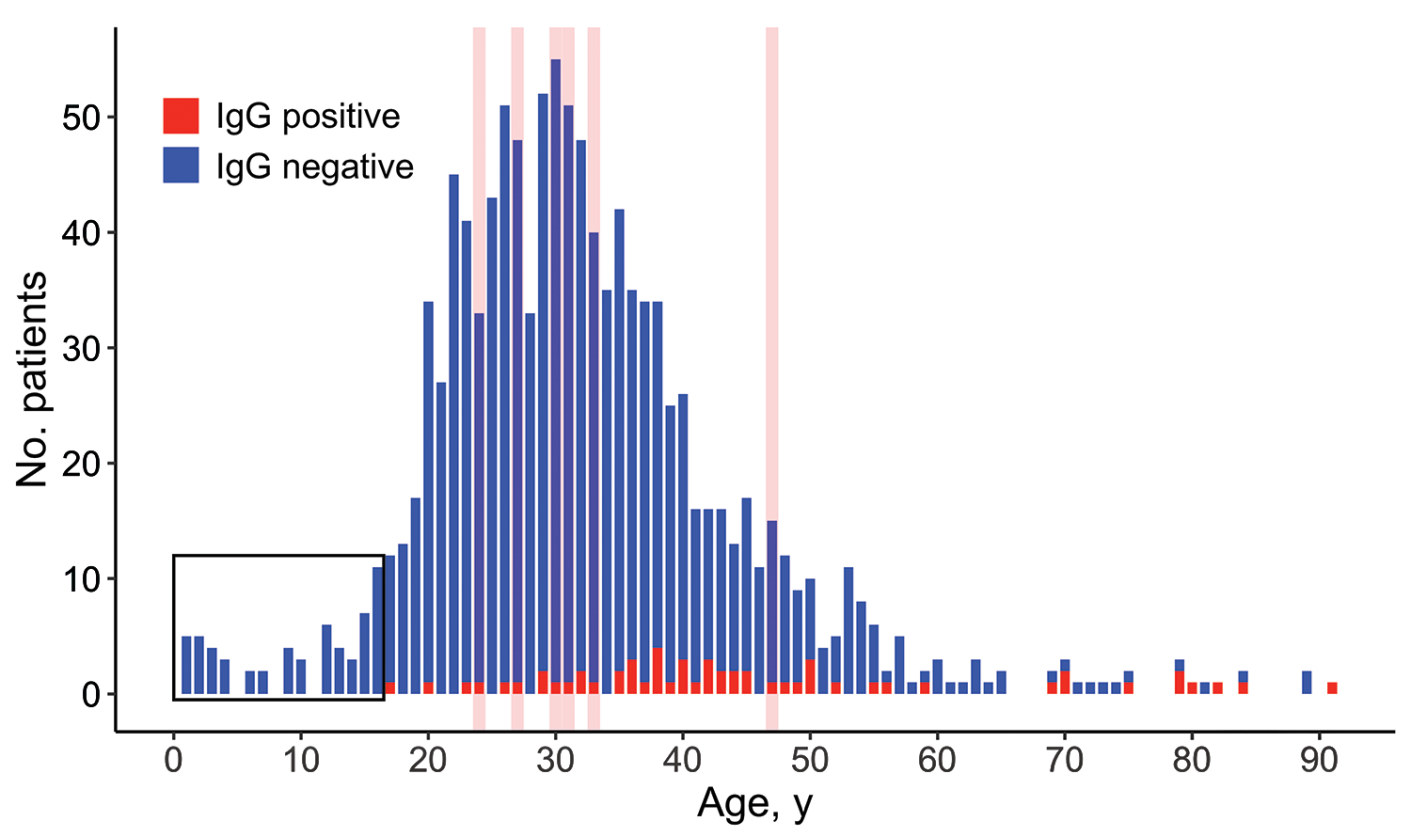
Number of Rift Valley fever virus IgG-positive IgG-negative human serum samples by age at time of testing, Botswana. The overlaid red lines represent ages of patients who also tested positive for Rift Valley fever virus IgM. No patients <17 years of age tested IgG positive for Rift Valley fever virus (black outline).

We found no reports of RVFV infections, confirmed or suspected, from Botswana’s passive health surveillance systems. Despite this, 5% (95% CI 4%–6%) of serum samples tested positive for IgG (mean age 46 [SD +17], range 17–91; Table 1); of these, 11% (95% CI 5%–21%) were positive for IgM (mean age 32 years [SD +8] range 24–47). Both IgG- and IgM-seropositive samples were found across sampled years, but no significant differences could be detected by year of testing (IgG and IgM χ^2^ = 0.27; p = 0.60) or season (seasonal data for IgG only available for 2013; χ^2^ = 0.98; p = 0.32). All IgM-positive samples (n = 7), however, were obtained during the wet season (November 2013–February 2014). During the ensuing dry season, a RVF outbreak in livestock (n = 2 cows) in the Chobe enclave in July 2014 was reported to the World Animal Health Information Database. In northern Botswana, rainfall and flood height affect mosquito dynamics, with models showing *Culex pipiens* mosquitoes to be most abundant in December (*9*), corresponding to human RVFV serological data previously collected in the region (*5*). The presence of IgM-positive patients confirms that RVFV was actively circulating in humans in the Chobe District in 2013 and 2014, causing a single outbreak potentially associated with RVFV infection in both humans and livestock.

**Table 1:** Percent of Rift Valley fever virus IgG-positive human serum samples by sex and age group, Botswana*

Age group	Age, y	Women			Men			Unknown	
		No. patients	No. IgG positive (%, 95% CI)		No. patients	No. IgG positive (%, 95% CI)		No. patients	No. IgG positive (%, 95% CI)
Child	<12	15	0 (0, 0–20)		17	0 (0, 0–18)		2	0 (0, 0–66)
Adolescent	13–19	57	1 (2, 0–9)		9	0 (0, 0–30)		1	0 (0, 0–79)
Young adult	20–24	152	2 (1, 0–5)		26	1 (4, 1–19)		2	0 (0, 0–66)
Adult	25–44	538	24 (4, 3–7)		150	7 (5, 2–9)		25	0 (0, 0–13)
Middle-aged	45–64	78	7 (9, 4–17)		45	4 (9, 4–21)		4	1 (25, 5–70)
Aged	65–79	5	1 (20, 4–62)		10	5 (50, 24–76)		1	0 (0, 0–79)
Elderly	>80	5	2 (40, 12–77)		3	2 (67, 21–94)		0	NA
Unknown	NA	11	1 (9, 2–38)		6	2 (33, 10–70)		114	5 (4, 2–10)
Total		861	38 (4, 3–6)		266	21 (9, 6–14)		149	6 (4, 2–9)
*NA, not applicable									

In South Africa, large RVFV outbreaks have occurred every 20–30 years, and domestic livestock has been implicated in human transmission (*10*). In Botswana, no human infections have been recorded, nor have explosive outbreaks occurred, suggesting that the dynamics of RVFV transmission and persistence differ between these countries. This difference may be because of differing agricultural production intensities and livestock composition; the Chobe District solely supports subsistence farming and has fewer small domestic ruminants. 

Overall, findings significantly differed by sex; men (n = 266, 9%, 95% CI 6%–13%) had higher IgG seroprevalence than women (n = 861, 4%, 95% CI 3%–6%) (χ^2^ = 4.96, p = 0.03; sex unknown, n = 149; Table 1). In contrast, all IgM-positive patients were female, except for 1, for whom sex was unknown. Sex-specific roles in animal care and food preparation might influence RVFV exposure patterns. In the Chobe District, 54% (95% CI 46%–61%) of interviewed households owned livestock (*11*), and men predominately cared for (97%, 95% CI 91%–99%; K.A. Alexander et al., unpub. data) and slaughtered large livestock (*12*). It is unknown why only women were IgM positive; however, women are involved in handling butchered meat in food preparation (*13*), so differences in exposure times to potentially infected animal tissues and fluids likely influenced RVFV exposure and transmission risk. Women (46%, 95% CI 32%–61%) and men (54%, 95% CI 39%–68%) both care for small livestock (K.A. Alexander et al., unpub. data). 

When we sorted patients into 7 age groups, elderly (>80 years old) and aged (65–79 years old) patients had significantly higher seroprevalence levels than other age groups (Table 2). A significant difference was also detected between middle-aged (45–64 years old) patients and young adults (20–24 years old) (Table 2), possibly because older patients have been exposed more often to RVF outbreaks as a function of time. However, low sample sizes in the aged and elderly groups may have skewed our results. All IgM-positive patients were 24–47 years of age. We found no evidence of RVFV in patients <17 years old, likely because of a lack of exposure to diseased animals (Figure). 

**Table 2:** Comparison of Rift Valley fever virus IgG prevalence by age group, Botswana*

Age group	Age, y	%, 95% CI	Adolescent	Young adult	Adult	Middle-aged	Aged
Adolescent	13–19	2 (0–8)	NA	NA	NA	NA	NA
Young adult	20–24	2 (0–5)	1.00	NA	NA	NA	NA
Adult	25–44	5 (3–6)	0.404	0.158	NA	NA	NA
Middle-aged	45–64	9 (5–15)	0.0814	**0.008**	0.07	NA	NA
Aged	65–79	40 (20–64)	**<0.001**	**<0.001**	**<0.001**	**0.007**	NA
Elderly	>80	50 (22–78)	**<0.001**	**<0.001**	**<0.001**	**0.01**	0.7
*Bold indicates significance (p value <0.05 by χ² test). NA, not applicable.							

Patient visits were primarily for routine health checks or noninfectious disease treatment, suggesting that RVF can occur with only mild or subclinical manifestations in affected people, which concurs with reports from other RVF-endemic regions (*13*). However, some IgG-positive patients in our study did have symptoms possibly attributable to RVF infection, including leg paralysis, swollen legs, and arthritis. Pregnancy was reported in 3 IgM-positive patients (43%, 95% CI 16%–75%); the outcomes of these pregnancies are unknown, but previous studies indicate that women infected with RVFV are 7 times more likely to miscarry than uninfected women (*14*). 

Where data were collected, we found a significant association between IgG seroprevalence and HIV status (χ^2^ = 6.4; p = 0.01); 48% (95% CI 36%–61%) of IgG-positive patients were also HIV positive. It is unknown when these patients became infected with RVFV or HIV, but concurrent infection can increase the development of RVF symptoms involving the central nervous system, as well as fatality rates (*15*). Although nearly one third of IgM-positive patients were infected with HIV (29%, 95% CI 8%–64%), we could not detect a significant association with HIV status (χ^2^ = 0.67; p = 0.4), likely because of the small sample size. 

## Conclusions

RVFV appears to be endemically circulating in northern Botswana, with people likely exposed to the virus regularly over time. Whereas viral reservoirs are uncertain, both livestock and wildlife present potential opportunities for human exposure to RVFV. In Botswana, government hospitals use syndromic diagnoses to treat patients; however, because no human cases have been reported and the disease can be asymptomatic, many RVF cases are likely misdiagnosed or undiagnosed. Increased diagnostic capacity and public health awareness of RVFV in Botswana is required to further elucidate risk factors associated with human infection, especially in high-risk populations. These findings underscore the urgent need for more intensive investigations into RVFV transmission and persistence at the human-animal vector interface. 
